# A model-based prion vaccine protects a transgenic mouse line carrying a Gerstmann–Sträussler–Scheinker disease mutation

**DOI:** 10.1007/s00401-026-03015-4

**Published:** 2026-04-17

**Authors:** Andrew Fang, Xinli Tang, Madeleine Fleming, Brian Tancowny, Xiongyao Wang, YongLiang Wang, Nathalie Daude, Lyudmyla Dorosh, Shelaine C. Fleck, Vineet Rathod, Virginie Coustou, Silvia A. Cervantes, Camilo Duque Velásquez, David Westaway, Judd Aiken, Debbie McKenzie, Glenn Telling, Maria Stepanova, Sven J. Saupe, Ansgar B. Siemer, Holger Wille

**Affiliations:** 1 Department of Biochemistry, University of Alberta, Edmonton, AB T6G 2R3 Canada; 2 Centre for Prions and Protein Folding Diseases, University of Alberta, 110C Brain and Aging Research Building (BARB), Edmonton, AB T6G 2M8 Canada; 3 Department of Electrical & Computer Engineering, University of Alberta, Edmonton, AB T6G 1H9 Canada; 4 Institut de Biochimie et de Génétique Cellulaire, Centre National de La Recherche Scientifique, IBGC, UMR 5095, 33077 Bordeaux, France; 5 Zilkha Neurogenetic Institute, University of Southern California, Los Angeles, CA 90033 USA; 6 Department of Biological Sciences, University of Alberta, Edmonton, AB T6G 2R3 Canada; 7 Neuroscience and Mental Health Institute, University of Alberta, Edmonton, AB T6G 2E1 Canada; 8 Department of Agriculture, Food and Nutritional Science, University of Alberta, Edmonton, AB T6G 1C9 Canada; 9 Department of Microbiology, Immunology, and Pathology, Colorado State University, Fort Collins, CO 80523 USA; 10 Present Address: School of Materials Science and Engineering, Harbin Institute of Technology, Weihai, Shandong China; 11 Present Address: Department of Cell Biology and Anatomy, University of Calgary, Calgary, AB T2N 1N4 Canada; 12 Present Address: Department of Chemistry and Biochemistry, University of Lethbridge, Lethbridge, AB T1K 3M4 Canada

**Keywords:** Prion vaccine, Protein engineering, Epitope prediction, Structure-based vaccine, Molecular modeling, GSS

## Abstract

Prion diseases are transmissible, fatal, neurodegenerative disorders driven by the conformational misfolding of the cellular prion protein (PrP^C^) into an infectious, aggregation-prone conformer (PrP^Sc^). While the accumulation of PrP^Sc^ represents the central pathogenic event, targeting it directly and specifically has proven difficult due to its structural heterogeneity and similarity to PrP^C^. Consequently, previous immunization efforts have largely focused on PrP^C^, though with limited success. Here, we employed a model-based approach to design a vaccine that specifically mimics immunogenic features hypothesized to be present on the surface of PrP^Sc^. By mimicking predicted surface residues and using an innocuous, cross-beta fibril scaffold derived from the fungal protein HET-s (218–289), we were able to generate unique, conformation-dependent epitopes not found on PrP^C^. We evaluated our vaccine by immunizing a genetic prion disease mouse model of Gerstmann–Sträussler–Scheinker disease, a genetic prion disorder. Vaccination was able to significantly delay the onset of disease in immunized mice (412 ± 88 days) compared to unimmunized (177 ± 17 days) and scaffold-immunized (161 ± 27 days) animals, and supplementation with adjuvants including Freund’s adjuvant (448 ± 39 days), QS-21 (479 ± 58 days), and Alum (506 ± 52 days) was able to further enhance the vaccine efficacy. To investigate the mechanism of neuroprotection, we derived a monoclonal antibody from a vaccinated mouse and mapped its discontinuous, conformation-specific epitope, comprising an aspartate–histidine pair that may be occluded in PrP^C^, and confirmed the antibody’s ability to differentiate between infectious and uninfected prion samples. Our study demonstrates the feasibility of a model-based approach for prion vaccine design and targeting of the infectious prion protein, providing groundwork for future development of not only potential prion therapeutic interventions, but also targeting related neurodegenerative disorders characterized by protein misfolding.

**Supplementary Information:**

The online version contains supplementary material available at 10.1007/s00401-026-03015-4.

## Introduction

Prions are self-replicating, infectious proteins that cause fatal neurodegenerative disorders including scrapie in sheep [39], bovine spongiform encephalopathy (BSE) in cattle [61], chronic wasting disease (CWD) in cervids [65], as well as Creutzfeldt–Jakob disease (CJD) and Gerstmann–Sträussler–Scheinker disease (GSS) in humans [40]. The misfolding of the mostly α-helical, cellular prion protein (PrP^C^) into the mostly β-strand containing, infectious prion protein (PrP^Sc^) results in a conformational change in both the tertiary and quaternary protein structure. The heterogeneous, insoluble, and often fibrillar nature of PrP^Sc^ made it difficult to obtain high-resolution structural information. Recently resolved *ex vivo* PrP^Sc^ structures contain a parallel‐in‐register intermolecular β‐sheet (PIRIβS) fold [2, 15, 16, 24, 29, 30]. However, it has also been suggested, based on prior experimental data, to adopt a four-rung β-solenoid (4RβS) structure [51, 53, 57, 62, 64].

Prions from yeast and filamentous fungi have also been described, including HET-s, which is a protein involved in heterokaryon incompatibility, a form of self-/non-self-recognition to prevent unwanted resource sharing [44, 46]. Of note, fungal prions are functional amyloids and unrelated to disease-causing mammalian prions [12]. The prion forming domain (PFD) of HET-s (residues 218–289) [4, 26] in its prion state is protease resistant (like PrP^Sc^), capable of forming self-propagating fibrils in vitro, and structurally contains a two-rung β-solenoid fold (2RβS) [60].

Many treatment attempts using small molecules, nucleic acids, as well as passive and active immunotherapies have been tried against prion diseases. These strategies can be broadly grouped into three categories: substrate reduction (lowering cellular PrP^C^), conversion inhibition (inhibiting the conversion of PrP^C^ to PrP^Sc^), and degradation/clearance (removal of the toxic species e.g., PrP^Sc^). Due to the inability to cure these diseases, most attempts are prophylactic in nature, working best when applied early in disease progression or prior to infection.

Vaccination against prion diseases has been shown to exert limited prophylactic effect in certain rodent adapted prion models and has relied mostly on synthetic peptides or recombinant prion proteins as antigens [27, 38, 47, 49]. Due to the tolerance of self-antigens by the immune system, however, the immune response generated from prion vaccines often has low affinity, rendering many of them ineffective. Prevention of peripheral prion infection following exposure may stop prions from invading the central nervous system and causing disease, but immunization to target PrP^Sc^ has been challenging, with many vaccination trials failing to account for the structural differences between cellular and infectious prions. Due to the infectious nature of CWD in cervids as well as its zoonosis concerns [42], there have been efforts to contain and prevent its spread using vaccines, with Goni et al*.* [13] showing partial protection for white tailed deer using an attenuated Salmonella vaccine, while Wood et al. [67] observed accelerated onset of CWD in elk immunized against PrP^Sc^. These results demonstrate the need for new approaches in designing more efficacious treatments and prophylactic vaccines against prion diseases. We hypothesize that mimicking specific surface residues of PrP^Sc^ in a vaccine can delay or prevent the onset of disease. Our approach is based on the 4RβS model for the structure of PrP^Sc^ and uses a modified, 4RβS version of HET-s as a scaffold to present selected surface residues that are unique to PrP^Sc^ to overcome the lack of host immune response typically associated with immunizations targeting the prion protein.

## Materials and methods

### Preparation of plasmid DNA

**Fig. 1:**
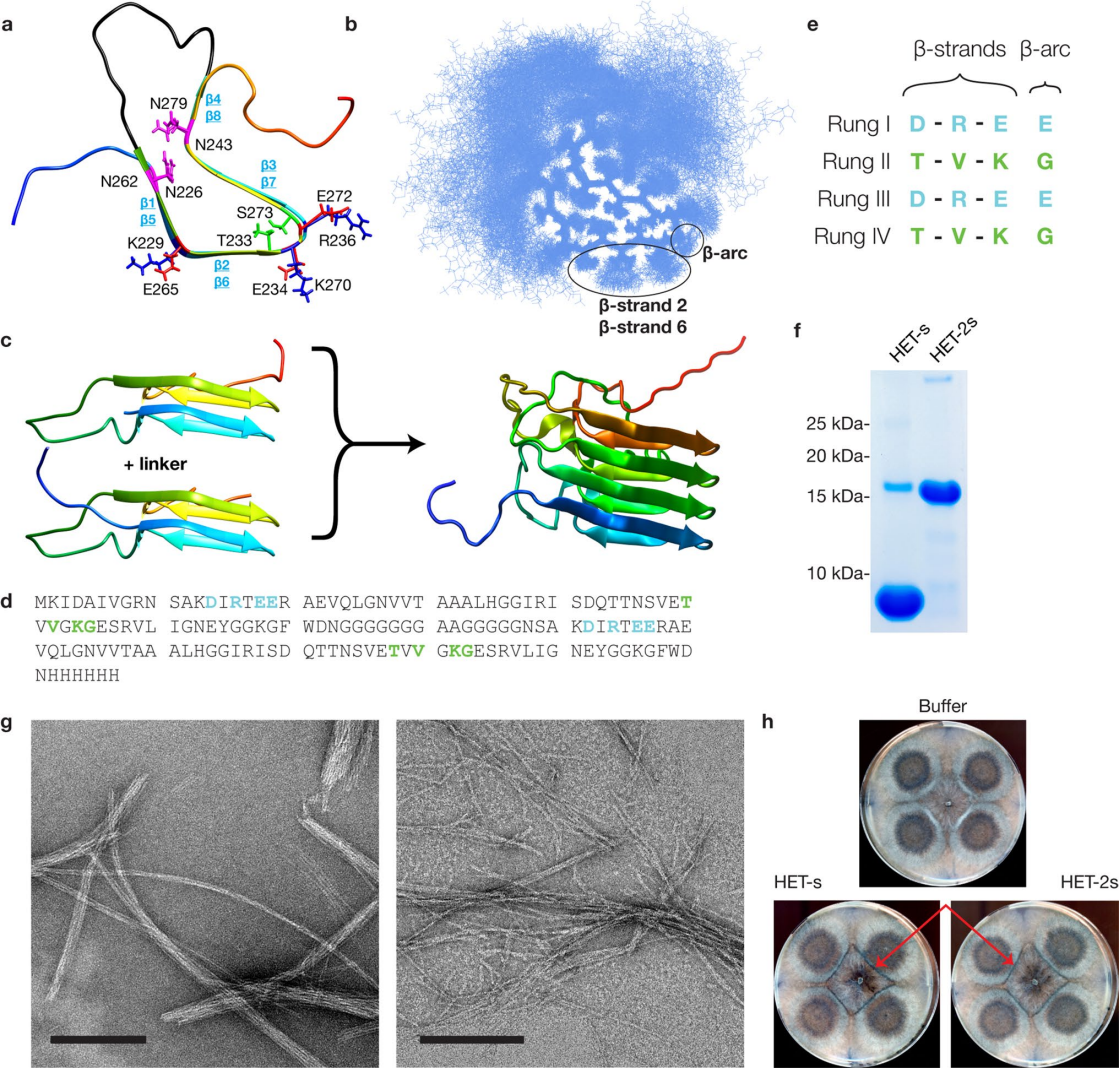
Fungal prion vaccine scaffold, HET-2s. **a** Structure of the C-terminal prion forming domain (PFD) of HET-s (residues 218–289) contains a triangular hydrophobic two-rung β-solenoid core consisting of 8 β-strands (teal). Backbone coloring runs blue (N-terminal) to red (C-terminal). Individual side-chain features of the β-solenoid include multiple salt bridges (red and blue, negative and positive salt bridge residues, respectively), two Asn ladders (purple), a 15-amino acid flexible loop (black) and two buried polar residues (green). (**b**) Top-down view of 100 HET-s PFD monomers superimposed, showing that residues from β-strands 2, 6 and the following β-arcs have surface-exposed residues. **c** Construction of HET-2s by connecting two HET-s PFD monomers (based on the structure determined by ssNMR spectroscopy, PDB: 2RNM (21) with a linker to create HET-2s, a 4RβS monomer (model assembled from the ssNMR structure). **d** Sequence of HET-2s; surface-exposed residues are indicated in cyan for rungs I/III and in green for rungs II/IV. **e** Array showing the surface-exposed amino acid residues of HET-2s, color-coded the same as (**d**) and corresponding to the regions in (**b**). Dashes indicate inward facing residues (not specified). **f** SDS-PAGE of purified proteins, showing a doubling of molecular mass from HET-s to HET-2s. **g** Negative staining electron microscopy of HET-s and HET-2s, depicting very similar fibril morphology. **h** Bioassay of HET-s and HET-2s in *Podospora anserina*, showing barrage lines (red arrows) on both constructs, which are absent in the buffer control. Scale bars for micrographs = 200 nm. Backbone coloring runs blue (N-terminal) to red (C-terminal) for (**a**), (**c**), and (**e**). (**c**) was prepared in ChimeraX. PDB for (**a**) and (**c**, left) is 2RNM

The nucleotide sequences were based upon an *Escherichia* *coli* (*E*. *coli*) codon-optimized version of the PFD of the fungal prion protein HET-s [26], spanning residues 218–289 [4]. Synthetic double-stranded DNA gene fragments containing 6× histidine tags were amplified with two universal primers with the following sequences: UniFwd (5’-GTCGTAGTCGCATAT GAAAATCGACGCTATTGTAGG-3’) and UniRvs (5’-TCGTCGTAGTCTCGAGTTAATGGT GATGATGATGGTG) using PCR. Amplified products were cloned into pET-17b vector via NdeI/XhoI and were sequence-verified with Sanger DNA sequencing. Linker constructs were optimized by connecting two monomers with reduced C-terminal glycines. Vaccine candidates were designed for HET-2s with either a 14 or 16 residue linker length and were constructed identically to the scaffold protein. Alternating surface residues on β-strands 2 & 6 were chosen as targets to be modified due to consistent surface amino acid exposure (Fig. 1b). Residue placement was loosely based on published threading work [50] and β-sheet conformation and stability [21]. Care was taken to ensure proper β-solenoid formation, resulting in the addition of salt bridges to ensure protein stability. Vaccine candidates all have a repetitive nature; rungs I and II are identical to III and IV, respectively, due to the previously mentioned salt bridge construction. Residues chosen were based on a human *PRNP* sequence ranging from residues ∼89–232. All additional constructs were created following the same steps.

### Protein production

Expression and purification of all constructs were performed using a modified method previously described [59]. All constructs were purified using the same protocol. Briefly, constructs were expressed as inclusion bodies in autoinduction media [52] and purified via lysozyme digestion, sonication and centrifugation. Purified inclusion bodies were then solubilized in denaturing conditions (50 mM Tris–HCl pH 8, 6 M Gdn–HCl) and clarified via ultracentrifugation, before being loaded onto Ni–NTA resin while maintaining denaturing conditions. Proteins were eluted via low pH (50 mM citric acid pH 2.0, 6 M Gdn–HCl), buffer-exchanged into 500 mM acetic acid, then titrated to pH 7.5 with 3 M Tris, and allowed to refold at room temperature. Sample concentrations were determined by BCA protein assay.

### Polyacrylamide gel electrophoresis

SDS–polyacrylamide gel electrophoresis (SDS-PAGE) samples were heated at 95°C for 5 min and separated on 12% Bis–Tris protein gels using MES running buffer for 35 min at 200 V. Gels were visualized using Coomassie G-250.

### Negative stain electron microscopy

Fibrillized samples at ~ 1 mg/ml concentration were adsorbed onto freshly glow-discharged, carbon-coated copper grids, washed with ammonium acetate, and stained with 1% filtered uranyl acetate. Grids were visualized using an FEI Tecnai G20 transmission electron microscope operating at 200 kV using a bottom-mounted Eagle 4K × 4K charge-coupled device camera at 19K or 29K magnification with a -0.50 to -1.50 μm defocus.

### NMR spectroscopy

Uniformly ^13^C-^15^N labeled HET-2s and YEG-Sc-1 fibrils were centrifuged into 1.6-mm nuclear magnetic resonance (NMR) magic angle spinning (MAS) rotors using a home-built packing tool. NMR spectra were recorded on a 600-MHz Agilent DD2 solid-state NMR spectrometer using a 1.6-mm triple-resonance probe. The sample temperature was set to 0°C. 2D ^13^C-^13^C DREAM (dipolar recoupling enhanced by amplitude modulation) spectra were recorded at a MAS frequency of 30 kHz and with a 4.5-ms-long adiabatic sweep through the HORROR condition. 140 kHz XiX decoupling was used during direct and indirect detection periods and 140 kHz continuous wave (cw) decoupling was used during mixing. Spectral widths of 50 kHz were used in both dimensions and for each of the 1000 indirect TPPI increments, 24 and 48 acquisitions were recorded for the HET-2s and YEG-Sc-1 fibril samples, respectively. Time domain data were processed with the nmrpipe and plotted using the nmrglue Python package.

### Protein bioassay

Protein transfection experiments of *Podospora anserina* with amyloid fibrils were carried out as described [5] with minor modifications. A [Het-s*] strain was cultivated for 24h at 26°C on corn meal agar. Three mycelial fragments (∼5 mm3) in STC buffer (0.8 M sorbitol, 50 mM CaCl2, 100 mM Tris–HCl pH 7.5) were supplemented with 100 μg of amyloids suspended in acetate buffer (424 mM acetic acid, 0.46 mM Tris) and then fragmented using a mechanical cell disruptor. Aliquots of the cell suspension were spotted onto corn meal agar plates inoculated at the center with a tester strain. After 4–5 d of regeneration at 26 °C, the plates were inspected for formation of a barrage reaction detected as a dark pigmented line in the confrontation zone between the mycelia.

### Immunogen preparation

Following confirmation of fibril formation and purity via negative stain electron microscopy and polyacrylamide gel electrophoresis, respectively, the sample was buffer-exchanged to phosphate-buffered saline (PBS) via centrifugation, tip-sonicated for 10 s at minimum amplitude at 4°C, and combined with the appropriate adjuvant. FAs were gradually added for a final 1:1 antigen:adjuvant ratio and emulsified until a single droplet no longer dissipated when placed on water [8]. Alum adjuvant was combined with the antigen at a 1:1 ratio to yield a 1 mg/ml alum concentration. QS-21 was resuspended in PBS and a 10:1 antigen:QS-21 ratio was used.

### Animal vaccination

The characterization of the GSS transgenic mouse line used in this study was previously described [33] with the mice designated Tg(GSS)22 and expressing a 12-fold normal level of PrP. In our facility, the animals were maintained on a hemizygous background, and were bred on a FVB/N background [54], and both male and female mice were used. All mice were monitored daily by animal technicians, including recording and scoring of clinical signs and making decisions for euthanasia of animals. The animal staff were blinded to the experimental groups.

Mice, between 6 and 8 weeks of age, were intraperitoneally immunized with a 100 μg antigen priming dose and 3 × 50 μg antigen booster doses every 2 weeks in a volume of 100 μl. Pre-immune sera were collected via tail bleeding 1 day prior to the priming dose, while subsequent collections occurred 13 days after injection (Fig. 3d). When using FAs as part of the inoculum for the initial efficacy trial, Freund’s complete adjuvant was used for the priming dose while Freund’s incomplete adjuvant was used for the boosting doses. Subsequent efficacy trials used QS-21 or Alum as adjuvants, or no adjuvant at all.

### Disease evaluation

The incubation period, or the time-to-disease onset, is the time between the date of birth and the first date on which definitive, subsequently progressive clinical signs were identified [41]. In addition to daily monitoring, the mice were assessed thrice weekly for the onset of neurological signs including progressive changes in behavior, aggression, coordination loss, grooming ability—which occurred throughout the course of disease—as well as a stiffened/rigid tail. Further, progressive symptoms included lack of righting reflex, head bobbing/tilting, circling, kyphosis (arched back), ptosis (droopy eyelids), balance issues, limb paralysis/dysfunction, dull or rough coat, tremors, weight loss, and blow test. Mice at the terminal stage of disease with 5 or more symptoms were humanely euthanized.

### Brain homogenates

Brain homogenates were re-suspended in radio-immunoprecipitation assay (RIPA) lysis buffer (Tris–HCl pH 7.4, 150 mM NaCl, 1% Nonidet P-40, 0.25% deoxycholic acid, 1 mM EDTA) with EDTA-free protease inhibitor cocktail to 10% weight/volume (w/v). Using an 18-gage blunt fill needle and 10 mL syringe, the brain-lysis buffer mixture was forcibly drawn and dispensed at least 50 times, until the mixture became a “homogenate”. BHs were then aliquoted and stored frozen at -80°C. For PK digestion of GSS samples, no protease inhibitors were added.

### Histopathology

For histology, collected samples were immediately immersed in 10% neutral buffered formalin for fixation. The samples then underwent a dehydration process and were paraffin-embedded. 4.5–6 μm sections were microtome-cut and collected on adhesive-treated slides and dried overnight at 37°C before being rehydrated via a xylene–ethanol–water process. Antigen retrieval was done in 10 mM citrate buffer at 121°C and 2.1 bar for 2 min. The slides were then stained with filtered Mayer’s hematoxylin, followed by eosin before being dehydrated in the reverse process. The slides were then covered with cover slips and dried at room temperature for 48 h.

Immunohistochemistry followed the same dehydration–rehydration–dehydration process as for the histology, with additional steps in the rehydrated state. The antigen retrieval for PrP^Sc^ detection was further enhanced by incubating the slides in 4 M guanidine thiocyanate. The biotinylated primary antibody was then added and detected with a secondary streptavidin peroxidase. DAB was then added until brown color was detected before being counterstained with Mayer’s hematoxylin. PrP^Sc^ and glial fibrillary acidic protein (GFAP) detection utilized SAF83 immunoglobulin G (IgG) at 1:500 and a mouse anti-GFAP IgG at 1:1000 dilution respectively. The rest of the procedure is as previously described for histology.

### Monoclonal antibody generation

After the last boosting dose, mice were given two more doses. The first dose contained 50 μg of antigen and Freund’s incomplete adjuvant, while the last dose contained only 100 μg of antigen. 3 days after the last dose, the spleens were collected and the splenocytes were disaggregated into a single-cell suspension via a 70 μm cell strainer. The isolated B cells were fused with immortalized myeloma cells using PEG in multi-well plates and cultured in HAT media to select for successfully fused cells. The supernatants from the clones were all screened against YEG-Sc-1 and HET-2s in an indirect ELISA. Clones showing reactivity toward YEG-Sc-1 and not HET-2s were further subcloned via limiting dilution. Repeated rounds of subcloning were performed to ensure monoclonality of hybridomas, and specificity against YEG-Sc-1 was validated before mass production.

For fragment antibody (Fab) generation, the purified monoclonal antibody G1 was dissolved in digestion buffer (20 mM sodium phosphate, 10 mM EDTA, freshly added 20 mM cysteine HCl pH 7.0) and was added to immobilized papain equilibrated in digestion buffer for digestion. The digestion reaction was incubated for five hours at 37°C on an end-over-end mixer. The digested product was collected by centrifugation and purified using an immobilized protein A column according to the manufacturer’s protocol. The digestion completeness of the IgG mAb and the purity of the Fab were assessed by SDS-PAGE.

### Enzyme-linked immunosorbent assays

All ELISAs utilized flat-bottom, high binding plates that were washed with TBS 0.1% Tween 20 (TBST). The secondary antibody was horseradish peroxidase (HRP) conjugated for cleavage of TMB substrate. Samples for coating were prepared identically as the immunization samples by buffer exchanging to PBS and sonication at minimum amplitude. For the peptide library, the samples were first re-suspended in DMSO before coating. The plates were coated with 0.5 μg antigen/well in 100 μl and incubated overnight before being blocked with 5% skim milk in TBST. For indirect ELISAs, the polyclonal mouse sera were serially threefold diluted in-plate starting at 1.00 × 10^–4^ dilution, while G1 was always added at 1.00 × 10^–4^ dilution. After 30 min of TMB cleavage in the dark, the reaction was stopped by the addition of sulfuric acid, and the optical density (OD) was then read at 450 nm (OD450).

The competition ELISA has one extra incubation step when compared to the indirect ELISA. Prior to the addition of the primary antibody, the competing antigen (e.g., BH) was diluted to 1 mg/ml and combined with the primary antibody 1:1 (v/v) and incubated in a non-binding plate for 1 h, before being transferred onto the antigen-coated plate. There is an inverse relationship between the level of enzymatically formed color change and the amount of antigen that was detected [28], which can be represented as a Δ OD value by subtracting the OD450 readout, with Prnp^−/−^ and uninfected BHs as the baseline readout subtraction and negative test control, respectively.

### Western blot

For protease digestion, the samples (15 μg, BCA assay) were treated with 1, 2, 10, and 50 μg/mL of PK for 60 min at 37°C, and the reaction was stopped by the addition of sample buffer. The samples were then subjected to SDS-PAGE as previously described at 110 V for 90 min. The gel was then semi-dry transferred to a PVDF membrane and blocked with 5% skim milk. The primary antibody, 38C12, is a lab-produced monoclonal antibody recognizing PrP residues 151–162. Membranes were then developed on film with an exposure time of 5 min.

### Immunogold labeling

Immunogold labeling was performed on purified RML PrP 27–30 samples as previously described [23, 56, 63] using the conformation-specific monoclonal YEG Sc-G1 antibody that recognizes a discontinuous epitope. Briefly, purified samples were adsorbed onto glow-discharged formvar/carbon-coated nickel grids, stained with sodium phosphotungstic acid (PTA), and blocked with bovine serum albumin (BSA). The grids were then incubated with G1 at 1 mg/mL, then with 6 nm gold-conjugated goat anti-mouse IgG, before being stained with PTA again. Wash steps were performed with TBS between all incubation steps. Control experiments were conducted similarly, except for the omission of the primary antibody.

The samples were analyzed using a Tecnai G20 transmission electron microscope as previously described. Gold particles that were observed within a radius of 26 nm from a structure of interest were considered specifically bound, since the length of an IgG (primary and secondary antibodies) molecule is 10 nm (10 nm primary YEG Sc G1 + 10 nm IgG + 6 nm gold particle diameter = 26 nm). Gold particles that exceeded this 26 nm distance threshold were deemed non-specific background labeling. 

### Structural threading

Structural models of the vaccine candidates were generated via structural prediction by submitting amino acid sequences of vaccine candidates with inserted prion residues to a profile-profile comparison algorithm [20, 45]. The resulting threading model was based upon various PDB entries and was produced using the UCSF Chimera package from the Resource for Biocomputing, Visualization, and Informatics at the University of California, San Francisco (supported by NIH P41 RR-01081) [18].

### Statistical analysis

Unpaired and paired t tests were performed for group- and pair-wise differences, respectively. Percent healthy and survival data were plotted in Kaplan–Meier curves and log-rank (Mantel–Cox) tests were performed for statistical significance. Ordinary one-way analysis of variance (ANOVA) using Tukey’s multiple comparison tests was performed when 3 or more sample types were involved. *p *values of < 0.05 were considered significant, and are listed as *, **, ***, ****, corresponding to *p *values of < 0.05, < 0.01, < 0.005, < 0.001, respectively. All analyses were performed using GraphPad Prism (GraphPad software Inc., version 10.2.0).

## Results

### A 4RβS version of HET-s as a protein scaffold

Due to the lack of high-resolution PrP^Sc^ structures when this project was started, we assumed that the 4RβS model would represent the main structural component of infectious prions. We selected HET-s as a scaffold due to the similarities between its 2RβS structure and the previously proposed 4RβS model of PrP^Sc^. The fibrillar HET-s core is mostly hydrophobic with salt bridges on the exterior, both serving to stabilize its amyloid structure (Fig. 1a). Superimposition of HET-s monomers revealed that β-strands 2 and 6 and the following β-arcs are surface-exposed, making these surface residues ideal targets for replacement (Fig. 1b). Two HET-s monomers were connected through a flexible, optimized glycine linker (Fig. S1) to create a 4RβS version, termed HET-2s (Fig. 1c, d). Thus, HET-2s contains twice the number of surface residues for manipulation spread over its four rungs (Fig. 1e). Polyacrylamide gel electrophoresis (PAGE) revealed a purified protein that is roughly twice the mass compared to the HET-s PFD (Fig. 1f), while transmission electron microscopy (TEM) displayed fibrils that were similar in general appearance, length, and abundance (Fig. 1g). Interestingly, fibrillized HET-2s remains a functional amyloid, capable of inducing the formation of barrage lines in *Podospora anserina* cells when supplemented in vivo (Fig. 1h).

### Design of YEG-Sc-1 as a prion vaccine

**Fig. 2:**
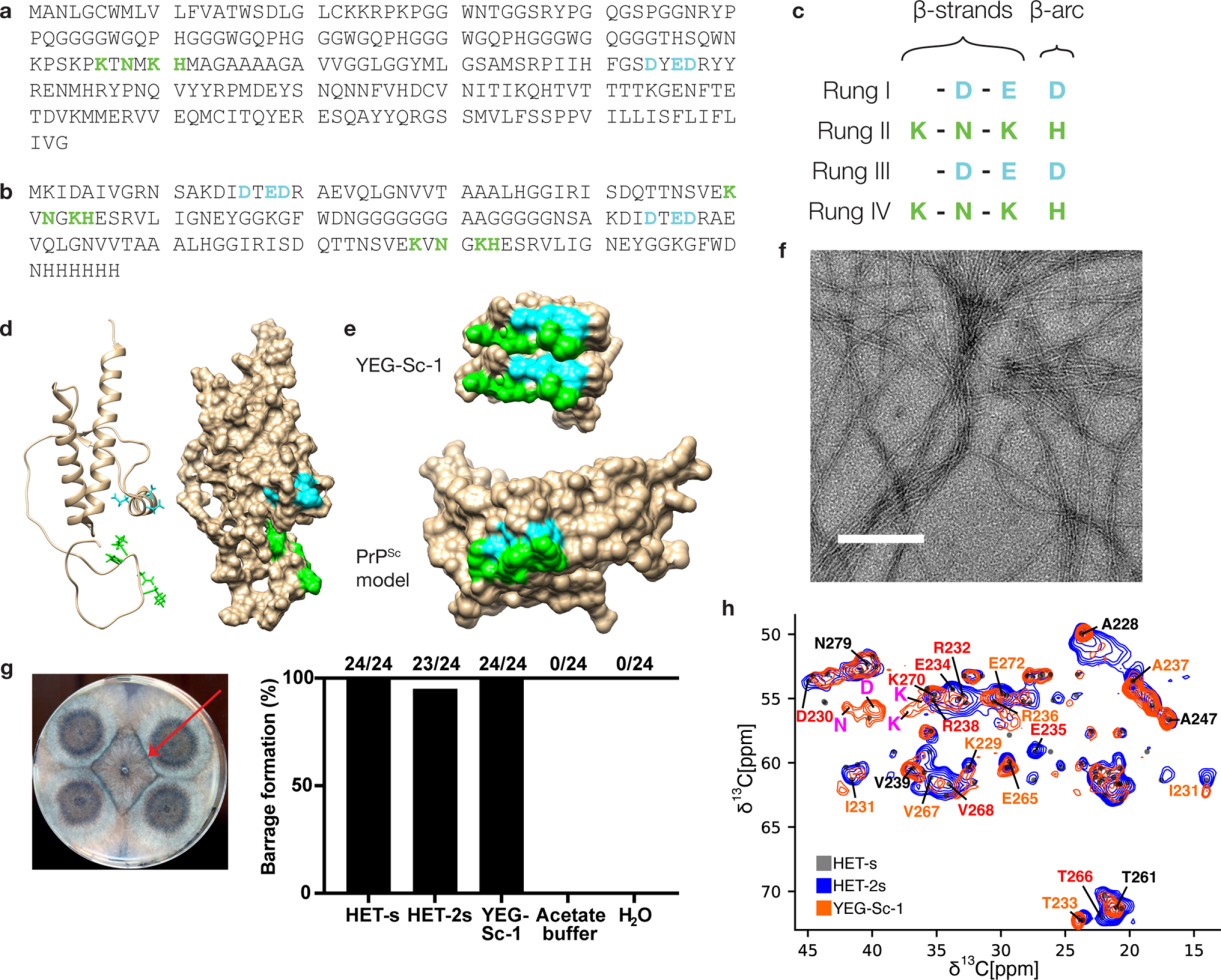
Prion vaccine YEG-Sc-1. **a** Sequence of the human prion protein. Residues chosen for surface replacement on the scaffold, color-coded the same as Fig. 1d, e. **b** Sequence of YEG-Sc-1. Surface-exposed residues from the β-solenoid model of PrP were inserted into HET-2s to generate YEG-Sc-1, color-coded the same as Fig. 1d, e. **c** Grid array of prion amino acid residues on the surface-exposed portion of HET-2s, using the same color and layout conventions as in Fig. 1e. **d** Ribbon and space-filling structures of human PrP^C^ adapted from Zheng et al. [69]. PrP amino acids highlighted in cyan correspond to residues on rung I/III and are found in the unstructured region while green highlighted residues correspond to rung II/IV and are found on helix 1, showing a discontinuous surface representation. **e** Space-filling models of the same, selected residues as in **c** on HET-2s and a PrP^Sc^ model [51], both showing a continuous surface epitope. **f** Negative staining electron micrography of YEG-Sc-1, showing similar fibrils as previously seen with HET-s and HET-2s. **g** Bioassay of YEG-Sc-1 in *Podospora anserina*, showing barrage lines (red arrow). **h** Overlay of solid-state NMR DREAM spectra of HET-s (gray, no contours), HET-2s (blue), and YEG-Sc-1 (orange). Residues modified in YEG-Sc-1 are highlighted in red, residues adjacent to a modification are shown in orange, and a few residues that are not close in sequence to an insertion site are highlighted in black. Peaks observed only in YEG-Sc-1 (orange) are also labeled in purple. Separated graphs in Fig. S5. Scale bars for micrographs = 200 nm. (**c**) and (**d**) were prepared in ChimeraX. The coordinate file for (**e**) is based on S1 [51]

Following successful construction of HET-2s, vaccine candidates were created using this scaffold with various surface residue replacements. The purification protocol involves denaturation and refolding of the protein, but the initial candidates were unable to refold into their native, fibrillar state and therefore would not form the designed epitopes (table S1). To increase proper folding, we opted to repeat the surface residues of a single HET-s monomer, thus increasing the number of salt bridges that would form. YEG-Sc-1 was created by grafting 7 predicted surface residues of PrP^Sc^ onto the surface-exposed region of HET-2s (Fig. 2a, b, c). These residues are discontinuous in PrP^C^, but in the 4RβS model of PrP^Sc^ they form a continuous, surface-exposed epitope (Fig. 2d, e). Incidentally, the selected surface residues are also discontinuous in the published PIRIβS structures of PrP^Sc^ [2, 15, 16, 24, 29, 30]. Purified YEG-Sc-1 fibrillizes into structures similar to HET-2s (Fig. 2f) and maintains its functional amyloid state as demonstrated in the fungal bioassay (Fig. 2g). Solid-state nuclear magnetic resonance (ssNMR) analyses further confirm YEG-Sc-1’s structure and proper epitope exposure as designed, with the modifications resulting in both missing peaks and a few new peaks not seen in the HET-s or HET-2s spectra (Figs. 2h and S2).

### YEG-Sc-1 delays disease onset

**Fig. 3:**
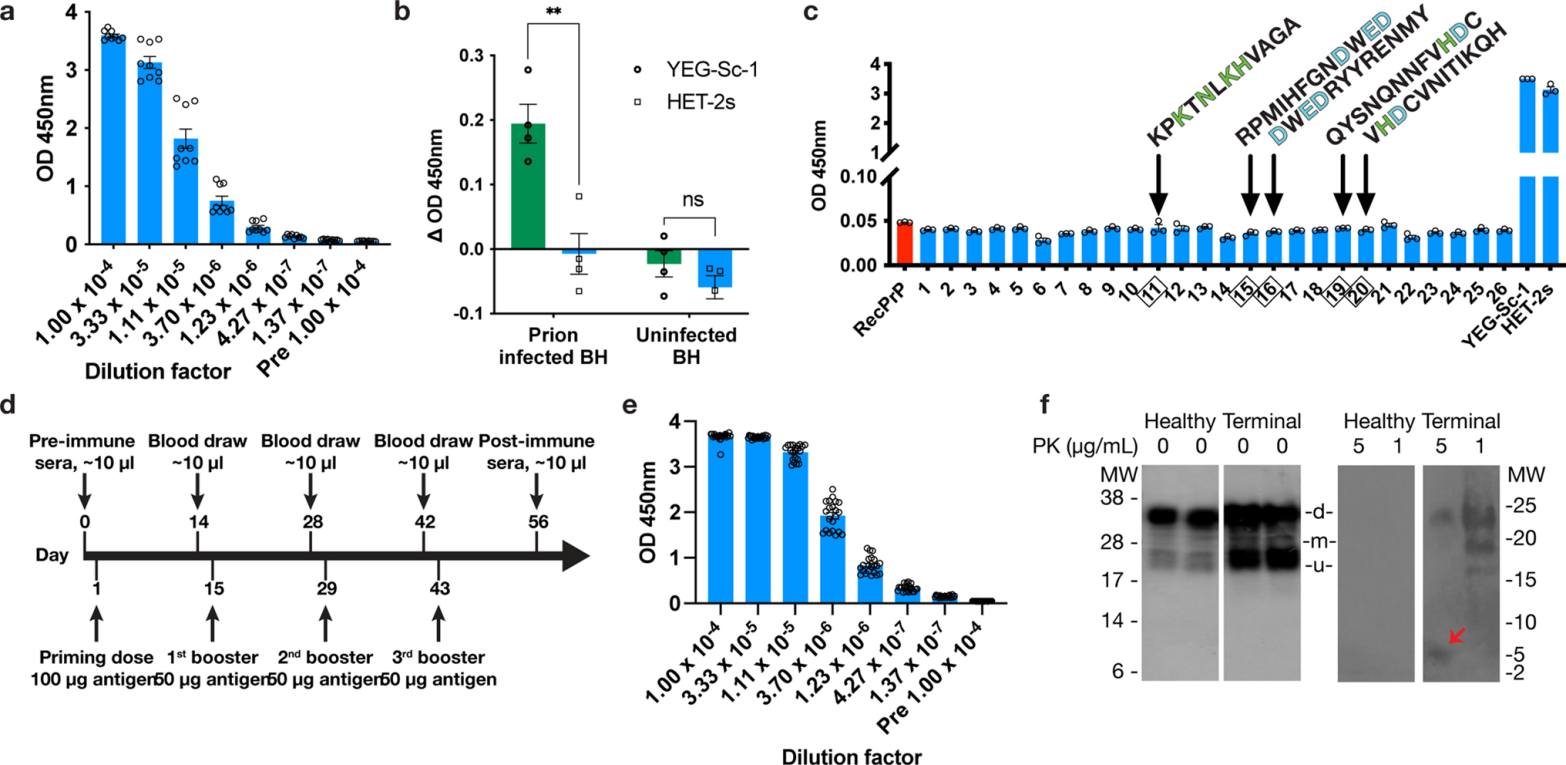
Immune response of YEG-Sc-1. **a** Indirect ELISA of serial threefold dilutions of post-immune sera from vaccine immunized wild-type mice. Each dot represents a technical replicate. **b** YEG-Sc-1 (green bars, circle dots) and HET-2s (blue bars, square dots) post-immune sera recognition of prion-infected brain homogenate (*p* = 0.0036) and uninfected brain homogenate (*p* = 0.2282) when used as the primary antibody in a competition ELISA (unpaired t tests). Each dot represents the average technical triplicate value of an individual sample. **c** Post-immune sera recognition of mouse PrP peptides in an indirect ELISA. Peptides 11, 15, and 16 contain the YEG-Sc-1 surface residues in a linear fashion, with the same color scheme as Fig. 2b, c. Peptides 19 and 20 contain a linear histidine–aspartate pair (HD), with the same color scheme. Recombinant PrP is highlighted in red. Each dot represents a technical replicate. **d** Vaccine immunization schedule with 1 priming dose and 3 boosters every 2 weeks and blood draws to obtain anti-sera. **e** Indirect ELISA of serial threefold dilutions of anti-sera from vaccine immunized TgP101L mice. Each dot represents a technical replicate. **f** Immunoblot of healthy and terminal TgP101L mouse brains. Terminal animals displayed significantly increased PrP levels compared to healthy animals. PK digestion products of healthy animal brains were unable to be detected while terminal animal brains yielded faint bands of weakly PK-resistant peptides at ~ 6 kDa (red arrow). Glycosylation levels are labeled, with d, m, and u corresponding to diglycosylated, monoglycosylated, and unglycosylated, respectively. Data are represented as mean ± SEM

**Fig. 4:**
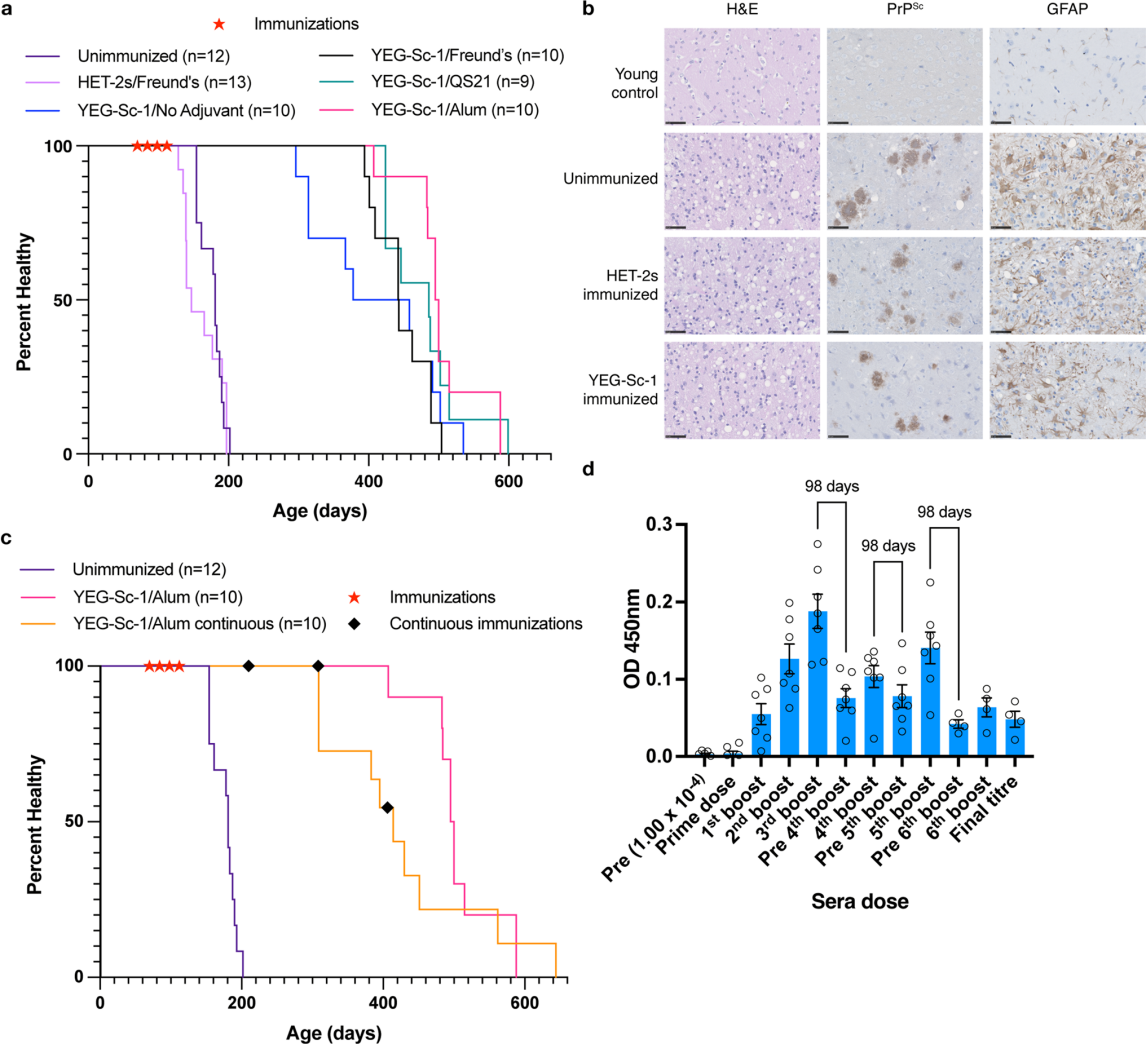
Vaccine efficacy of YEG-Sc-1 in TgP101L mice. **a** Kaplan–Meier curves depicting the health of mice with YEG-Sc-1 and FAs (black line), QS-21 (green line), Alum (pink line), or no adjuvant (blue line) compared to unimmunized (purple line) and HET-2s/Freund’s (light purple line). Animals were immunized (red stars) on a prime-boost schedule as described in Fig. 3d. Summary statistics in Table S2. **b** Histopathology analysis of terminal mouse brains. Representative images of H&E, GFAP and PrP^Sc^ staining from each animal group. Scale bars = 50 μm. **c** Kaplan–Meier curves depicting the health of mice immunized with YEG-Sc-1/Alum on the regular prime-boost schedule (pink line, red stars, data copied from panel **a**) and continuous boosts (orange line, black diamonds) compared to unimmunized mice (purple, data copied from panel **a**). Summary statistics in Table S3. **d** Indirect ELISA using sera at a 1.37 × 10^–7^ dilution from continuously immunized mice

To enhance the immune response of our vaccine, we utilized Freund’s adjuvant (FA) as part of the immunogen. We initially immunized a small number of FVB wild-type mice by intraperitoneal (IP) injection to ensure vaccination was well tolerated by the animals and to test the vaccine immunogenicity. YEG-Sc-1 was indeed well tolerated and immunized mice developed robust titers against the antigen (Fig. 3a). The polyclonal anti-sera, when used as the primary antibody in a competition enzyme-linked immunosorbent assay (ELISA), were able to differentiate between prion-infected and -uninfected brain homogenates (BHs) (Fig. 3b). The anti-sera were unable to recognize linear prion protein (PrP) peptides, suggesting they recognized a discontinuous, structured epitope (Fig. 3c). Immunization and testing of anti-sera from other vaccine candidates did not elicit this same PrP^Sc^-specificity as seen with YEG-Sc-1 (Fig. S3). After confirming the specificity of the immune response, we IP immunized transgenic (Tg) mice that spontaneously develop GSS based on a proline to leucine missense mutation at codon 101 (P101L) [33], which corresponds to the human P102L mutation [17] (Fig. S4). Mice were subjected to 1 priming dose (100 μg antigen) and 3 booster doses (50 μg antigen) along with FA in 2-week intervals, and subsequently developed very high antibody titers (Fig. 3d, e). Vaccine-immunized animals were asymptomatic for up to 448 ± 39 days while unimmunized and scaffold-immunized animals developed symptoms after 177 ± 17 and 161 ± 27 days, respectively (Fig. 4a). Due to toxicity concerns with using FA, we repeated the trials using alum and QS-21 as alternative adjuvants, as well as excluding the adjuvant entirely. All YEG-Sc-1 immunized animals, regardless of adjuvant (or lack thereof), remained healthy for significantly longer periods (Fig. 4a, Table S2, and Fig. S5). Histological examination of terminal mouse brains confirmed that all animals eventually developed prion disease pathology, regardless of immunization status, while young control animals show normal tissue morphology under H&E, and no PrP^Sc^ plaques or gliosis as represented by GFAP-staining (Fig. 4b). Unimmunized, HET-2s, and YEG-Sc-1 immunized animals all show typical spongiform change under H&E staining, as well as GFAP-dependent gliosis and PrP^Sc^ plaques, with corpus callosum (H&E), cerebral cortex (PrP^Sc^), and hippocampus (GFAP) regions represented. Immunoblotting of healthy and terminal brain samples required very low amounts of proteinase K (PK) due to the animals harboring PK-sensitive prions (Fig. 3f). We also continuously immunized mice with YEG-Sc-1 and alum, with these animals developing clinical symptoms earlier than the prime-boost schedule previously utilized (Fig. 4c and Table S3). The antibody titers for these animals reached a maximum following 3 boosts, and subsequent boosts did not further increase this titer (Fig. 4d).

### Monoclonal antibody G1 recognizes a structural epitope

**Fig. 5:**
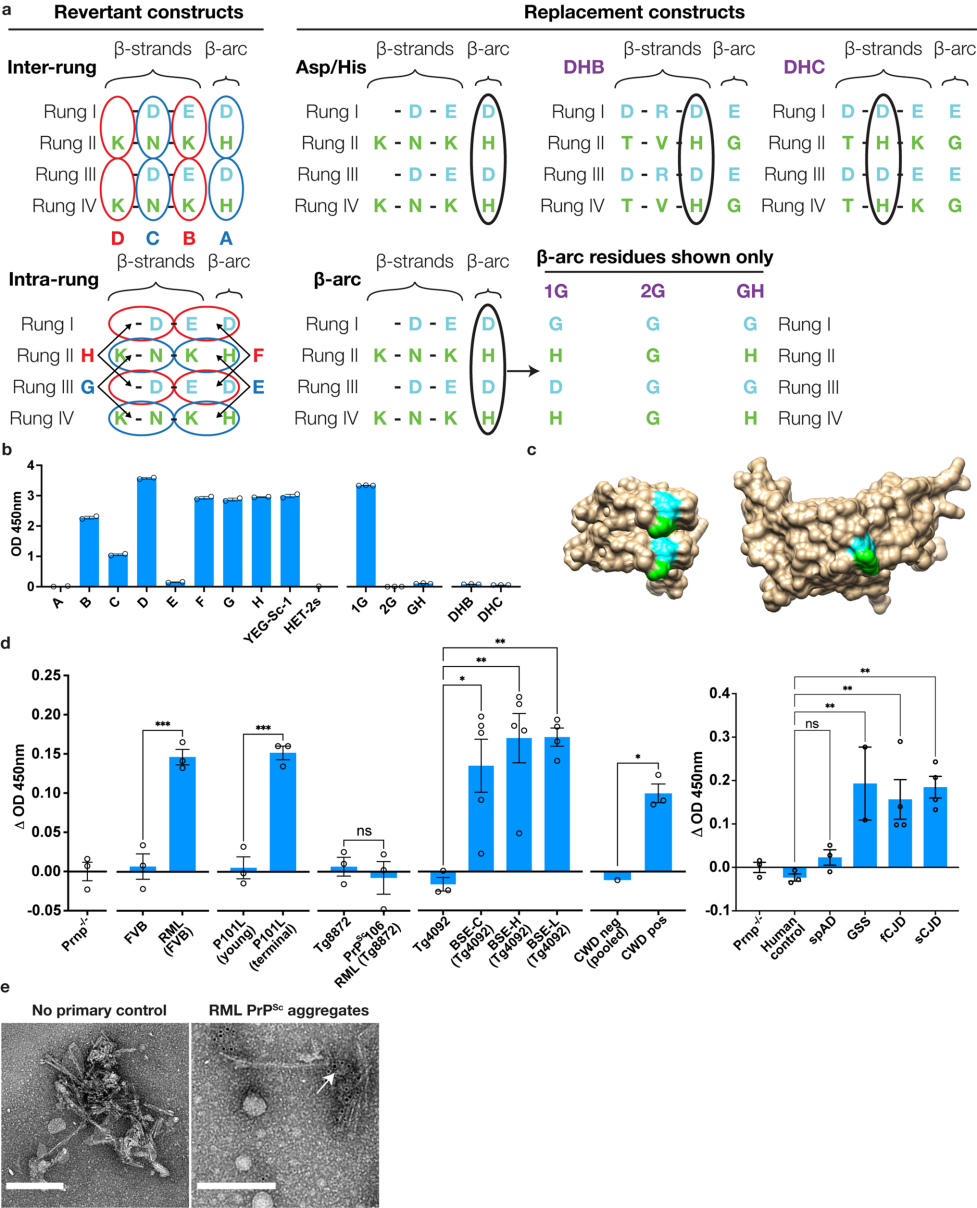
Epitope mapping of monoclonal antibody G1. **a** Schematic representation of revertant (left) and replacement (right) constructs necessary to determine the exact epitope residues recognized by G1, in the same color and layout convention as previously described. Inter- and intra-rung revertant constructs had pairs of residues (small blue or red ovals) reverted back to the original HET-2s residues, and are titled A–D and E–H, respectively. Asp/His replacement constructs moved the residues to β-strands from the β-arc (large black ovals), titled DHB and DHC (purple), while β-arc replacement constructs involved replacing either Asp or His (large black ovals) with Gly, titled 1G, 2G, and GH (purple). **b** G1 epitope mapping using the constructs from (**a**) in an indirect ELISA. Each dot represents a technical replicate. **c** The epitope of G1 is Asp and His on the β-arc and is highlighted in green and cyan, respectively, on YEG-Sc-1 (left) and the 4RβS model (right) from Spagnolli et al. [51]. **d** Competition ELISA of G1 Fab against human (right) and animal (left) prion strains and uninfected counterparts with Δ OD values. Each dot represents the average technical triplicate value of an individual sample. Sequence comparison of the species used here in Fig. S6. **e** Negative stain TEM micrographs of RML immunogold labeling using IgG G1, with white arrows indicating labeling of aggregates. Scale bars = 200 nm. All error bars indicate SEM. Statistical significance was calculated via ordinary one-way analysis of variance (ANOVA) [(**d**)] in GraphPad Prism. n.s. *P* > 0.05; *P < 0.05; **P < 0.01; ***P < 0.001

We generated monoclonal antibodies from YEG-Sc-1 immunized FVB wild-type mice that recognize only YEG-Sc-1 but not the unmodified HET-2s scaffold, thus potentially being able to recognize a structural epitope present on PrP^Sc^. The monoclonal antibody “G1” was selected after repeated subcloning to ensure monoclonality and verification of YEG-Sc-1-specificity. We then created a fragment antibody (Fab) version of G1 to eliminate bridge ELISA effects and tested the fragment G1 Fab in competition ELISAs against a variety of human and animal prion strains to verify its specificity toward infectious samples. G1 Fab was able to preferentially recognize human brain samples from patients with GSS, familial CJD (fCJD), and sporadic CJD (sCJD) over non-neurologic human controls and sporadic Alzheimer’s disease (spAD) samples (Fig. 5d). G1 Fab was also able to preferentially recognize mouse brain samples of different prion strains, e.g., sheep scrapie strain RML, all three BSE strains: BSE-C, -H and -L as well as the GSS P101L, and a CWD-positive deer sample over their non-infectious controls (Fig. 5d). G1 was unable to recognize PrP^Sc^106 RML samples (n.s.). IgG G1 recognized PrP^Sc^ aggregates but not highly purified fibrils in immunogold labeling visualized using TEM (Fig. 5e).

The rational design of the YEG-Sc-1 antigen allowed us to determine the structural epitope on the engineered vaccine and potentially on PrP^Sc^. We created four inter-rung (constructs A–D) and intra-rung (constructs E–H) mutants that cover the entire surface-exposed region of the vaccine by reverting residue pairs back to the original HET-2s scaffold residues and tested G1’s ability to recognize them (Fig. 5a). All mutants were recognized except constructs A and E, with both missing a histidine at the β-arc position of rungs II and IV. To confirm the epitope, we created three more constructs with variations of histidine and aspartate at the β-arc position only. G1 was able to only recognize construct 1G, containing both an aspartate and histidine pair, which were absent from the other two constructs (Fig. 5b). To determine the importance of residue positioning, we created two more constructs which moved the aspartate–histidine pair onto β-strands 2 and 6. However, G1 was unable to recognize any of these constructs, suggesting that the epitope is a discontinuous aspartate and histidine pair, specifically positioned at the β-arc position with its unique side chain restraints [22] (Fig. 5b, c).

## Discussion

In this study, we describe the creation of a model-based vaccine against prion diseases. This is contrasted with previous prion vaccine design attempts in which minimal consideration was given to the structural differences between PrP^C^ and PrP^Sc^ and which often relied on synthetic peptides of the prion protein. By mimicking the putative β-solenoid backbone with a compatible scaffold protein and placing specific residues on its surface, we were able to create a vaccine that resembles a previously proposed conformation of PrP^Sc^ (4RβS) but in a completely innocuous protein which has no homolog among vertebrates [12]. The resulting vaccine was able to generate strong antibody titers that we presume to be conformation-selective and that were consequently found to be capable of significantly delaying disease onset (all P values < 0.0001, table S2) in a Tg GSS mouse model [33]. The immunized mice also showed significantly increased survival versus unimmunized controls regardless of adjuvant (except for the HET-2s/Freund’s control, table S2). While the mice ultimately succumbed to disease, this could be explained by the advancing age of the mice or lymphocyte exhaustion, and the latter is also linked to amyloid accumulation in AD [14]. Lymphocyte exhaustion could also explain the counterintuitive earlier disease onset in mice that received additional YEG-Sc-1/alum boosters (Fig. 4c, d). However, given that the final neuropathological results are the same regardless of vaccination status, the precise mechanism by which the immunization delays the onset of the disease remains to be determined. A detailed time course study would be necessary to clarify this further. The unique design of our vaccine allowed us to create a monoclonal antibody that differentiates between prion-infected and -uninfected brain homogenate samples, and to provide insights into its discontinuous, structured epitope. This general method of vaccine and antibody development could be applied to other neurodegenerative diseases, such as Alzheimer’s disease and Parkinson’s disease. In the latter case, the protection that was observed with structure-based vaccines targeting misfolded α-synuclein may have interfered with the cell-to-cell spreading of the intracellular α-synuclein aggregates [11, 25, 36].

The use of a foreign protein scaffold to imitate a conformational antigen (i.e., PrP^Sc^) has several benefits: 1) the foreign nature of the protein is beneficial for inducing a strong immune response, 2) the protein is harmless to the host and will not cause prion disease, and 3) the immune response is specific for infectious prions. Using a modified, recombinant HET-s protein as the scaffold has further benefits, such as quick verification of proper epitope presentation (fibril formation), straightforward epitope determination and manipulation, and ease of production (yield and purity). This makes our approach unique when compared to prior vaccination attempts, where weaker immune responses were often observed [10, 34, 38, 49], and in a rare circumstance, accelerated onset of disease [67]. Furthermore, we determined the structured epitope of YEG-Sc-1, which is either impractical or impossible for most vaccines due to a lack of structural control. While vaccination against PrP^C^ is much more common than PrP^Sc^ [1, 9, 19, 37, 68], lowered PrP^C^ levels reduce its ability to perform its native functions, although substrate reduction itself appears to also be a viable approach for prion treatment [31, 32, 43]. Targeting infectious prions comes with its own challenges, mainly due to the insolubility of amyloid fibrils. Typical immunoassays detect PrP^Sc^ in its denatured, linear form, eliminating any structured epitopes for detection, and even without denaturation amyloid fibrils are typically too large for efficient and reproducible coating onto microplates. Competition ELISAs were utilized for several reasons; they can detect analytes within complex mixtures (e.g., brain homogenate), require minimal sample processing, and are able to detect antigens in a near-native environment. Thus, the competition ELISA format keeps PrP^Sc^ in its native state with minimal processing, preserving its structural integrity and allowing us to verify the specificity of our antibody.

To establish the conformational-selectivity of G1, a G1 Fab was generated for use in competition ELISAs, eliminating possible bridging effects in ELISAs associated with the bivalent nature of monoclonal antibodies [55]. G1 Fab was able to preferentially recognize a variety of animal and human prion brain samples over non-infectious controls. The limited number of GSS samples and the comparison of pooled CWD-positive and -negative brain homogenates precluded statistical analyses for these samples. Human spAD samples were not recognized by G1 Fab, serving as an extra control to demonstrate its prion specificity. Furthermore, the inability to recognize PrP^Sc^106 RML samples also supports the identified conformational epitope of G1 (Fig. 5d), based on the truncated nature of this mini-prion and the prion protein residues (141–176) that were deleted [53, 64], which may include the ones that are recognized by G1 Fab. Despite the incompatibility of our vaccine with the existing PIRIβS prion structures, all full-length, prion-infected brain homogenates used in this study were recognized over their non-infectious counterparts (prion protein sequence information in Fig. S6). Moreover, the overall competition ELISA results indicate that a conformational epitope that is present in infectious prion samples is being recognized by G1 Fab, which was ultimately derived from a 4RβS model. IgG G1 was able to recognize RML PrP^Sc^ aggregates but not mature fibrils (Fig. 5e); indeed, the current *ex vivo*, fibrillar PrP^Sc^ structures all present as PIRIβS structures [2, 15, 16, 24, 29, 30] that are distinct from the previously proposed 4RβS architecture [51].

The proposed mechanism for YEG-Sc-1’s prophylactic efficacy is based on the conformational recognition of a disease-specific antigen by antibodies or immune cells and the subsequent removal from the host. Due to the absence of a peripheral infection route in genetic prion diseases, the antibodies of the elicited immune response are assumed to cross the blood–brain barrier (BBB) to have an effect. It is widely accepted that the BBB limits the entry of both immune cells and immune mediators, making the brain a relatively immune-privileged site that has different immune responses than those in the periphery [66]. A way to get around this is to use receptor-mediated transport [35], but antibodies like Aducanumab [6, 48] or Lecanemab [3] are able to effectively clear Aβ plaques in the central nervous system without involvement of a transport system, showing that some antibodies are able to cross the BBB in sufficient concentrations. It is possible that the immune response elicited by YEG-Sc-1 works in a similar way and that the resulting antibodies are also able to penetrate into the brain parenchyma.

The clear differentiation between infected and uninfected brain homogenates with our G1 Fab (Fig. 5d) raises the question of how many PrP conformers can be found in an infectious sample. The ‘cloud hypothesis’ posits that each prion isolate or strain is composed of a multitude of conformers, which could explain our findings [7]. Our data indicate that the epitope of G1 contains a discontinuous aspartate and histidine pair on a β-arc position in the original YEG-Sc-1 antigen, which is presumed to exist in all prion strains that G1 recognized. The individual ΔOD values of the competition ELISAs are generally not very large, but this is likely the result of the limited quantity of PrP^Sc^ in this conformation recognizable by G1 Fab, since no enrichment or purification were performed. Whether this theoretical conformer exists in low relative amounts in the samples or G1 Fab exhibits low sensitivity, or a combination of both, is not known. The sensitivity could also be reduced via cofactors within samples (BHs), possibly interfering with the recognition by either masking the target epitope or non-specifically binding to G1 Fab itself. While our data clearly demonstrate that G1’s binding strictly requires an aspartate and histidine pair within the β-arc of the original YEG-Sc-1 antigen, the direct engagement of an identical, pre-formed discontinuous epitope on native PrP^Sc^ remains to be structurally validated. The evidence linking our engineered scaffold to the native conformation of PrP^Sc^ is necessarily indirect, and while it is highly consistent with the presence of a similar structural epitope in a subpopulation of PrP^Sc^, it does not constitute direct proof of the 4RβS architecture model our vaccine is derived from. Currently, published *ex vivo* prion structures are all PIRIβS-based, and closer examination shows that such a β-arc containing an aspartate and histidine pair is absent [2, 15, 16, 24, 29, 30]. A possible explanation for this discrepancy, despite the preferential recognition by G1 Fab, is the existence of intermediate folds that may exist on the pathway for many prion strains. Prion purifications often include harsh but necessary isolation steps that inherently enrich species that are highly protease- and detergent-resistant, while intermediate structures are often far less stable. This discrepancy does not invalidate the utility of our model; rather, it highlights the possibility that our antibody may recognize a minor, perhaps transient or oligomeric conformation that is immunologically present but structurally underrepresented in bulk preparations. It is then possible that the published PIRIβS structures represent an end-stage phenomenon, one that is highly stable and also infectious, but not the sole structural species that exists, again in agreement with the ‘cloud hypothesis’[7]. Purification of these intermediate structure subpopulations in quantities suitable for structural analysis is challenging, and likely not currently possible. Therefore, our findings are not a definitive validation of the 4RβS model, but rather the empirical generation of a conformation-selective vaccine with anti-prion responses whose binding characteristics (G1 Fab) can be explained by the 4RβS architecture model, and serve as a functional mimetic of a disease-relevant epitope.

Moreover, while G1 Fab demonstrates broad in vitro binding across a range of human and animal prion strains, including RML, BSE, CWD, and various CJD subtypes (Fig. 5d), this binding breadth does not necessarily predict equivalent therapeutic reach. The present study establishes proof-of-concept efficacy exclusively in the transgenic P101L model of GSS disease. We selected the P101L model because it represents a stringent, spontaneous model of prion neurodegeneration that develops end-stage disease in under ~ 200 days, enabling us to evaluate the therapeutic potential of our vaccine in a rapid, chronic, and progressive context that closely models an inherited human prion disease. In contrast, classical, experimentally inoculated prion strains are more representative of acute transmission events and given the usually high doses of prion inocula, they are less suited to capturing the spontaneous disease onset modeled here. However, GSS is biologically distinct, often involving truncated PrP^Sc^ fragments (~ 8 kDa), and, therefore, it is possible that the protective effect we observed depends on a specific conformer or subpopulation of PrP^Sc^ that is particularly abundant or accessible in this model. The translational potential of our approach in other prion strains will likely be governed by strain-specific variation in the conformation and accessibility of this epitope, whose structure remains to be confirmed. Therefore, it remains to be established whether the conformation-selective mechanism of the YEG-Sc-1 vaccine, which we have identified, is broadly conserved across prion diseases or is a feature unique to the P101L model.

Lastly, the broad, yet low-magnitude binding of G1 across multiple prion strains implies that the epitope is structurally conserved, but its presentation is likely restricted to a specific subset of PrP^Sc^ conformers within the total population, which may be present at low abundance only. Consequently, it is theoretically possible that sustained immunological pressure from active vaccination could select for a subpopulation of PrP^Sc^ conformers that lack the G1 epitope or present it in an inaccessible conformation. Such conformers could, in principle, propagate and give rise to a vaccine-resistant prion strain. While we observed no direct evidence of breakthrough cases, the experimental design was not optimized to detect the emergence of resistant conformers, and the possibility cannot be excluded. Future studies aimed at translational development should therefore include longitudinal monitoring for epitope loss or conformational switching, particularly in models of classical inoculated strains where prion diversity is well-documented. Determining whether the conformation-selective mechanism of YEG-Sc-1 is broadly applicable or susceptible to escape will be critical for assessing its long-term therapeutic potential.

In summary, efficacious prion prophylactics and treatments targeting PrP^Sc^ have been difficult to design, with other treatment regimens being either strain or disease-specific [58]. While factors, such as variations in immune systems in different species, exposure route and quantity, and specific genotype differences, can all influence the eventual prion phenotype, our approach may address this by selecting unique epitopes that are absent on PrP^C^, as well as minimizing other basic biochemical and technical challenges associated with typical prion vaccines as previously mentioned. While the prophylactic uses of the vaccine and the antibody for other prion diseases or strains will require further investigation, our approach can also be applied for prion structural analysis and assay development. Furthermore, the principles underlying model-based/structure-based vaccines can also be applied to other neurodegenerative diseases involving protein misfolding [11, 25, 36].

## Supplementary Information

Below is the link to the electronic supplementary material.

Supplementary file1 (PDF 3799 KB)

## Data Availability

Materials will be made available upon completion of a materials transfer agreement (MTA). All data are available in the main text or the supplementary materials.
