# Supplementary material for: A model-based prion vaccine protects a transgenic mouse line carrying a Gerstmann–Sträussler–Scheinker disease mutation
Source: Acta Neuropathol. 2026 Apr 17;151(1):41. doi: 10.1007/s00401-026-03015-4 (PMC13090231; doi:10.1007/s00401-026-03015-4)
Supplement: Supplementary file 1 — Supplementary file1 (PDF 3799 KB) [file 401_2026_3015_MOESM1_ESM.pdf]

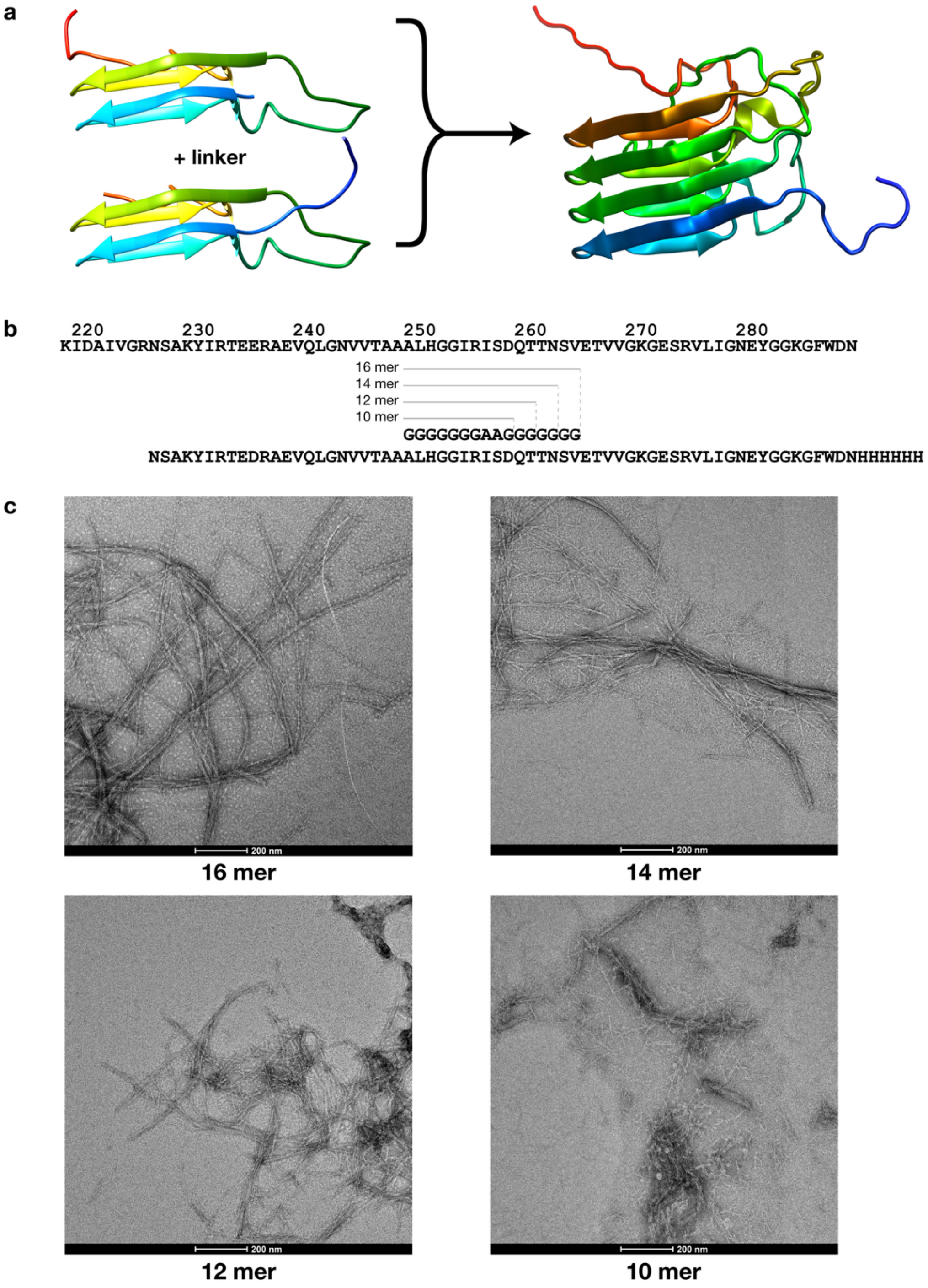

Fig. S1 Linker optimization of the HET-2s scaffold protein. **a** Two HET-s PFD monomers were connected with a linker to get HET-2s, a four-rung β-solenoid monomer. **b** Amino acid sequence of HET-2s with linkers ("mers") ranging from 16 to 10 residues. **c** Electron micrographs (recorded at 29K magnification) of HET-2s mers stained with 1% uranyl acetate, showing very similar fibril structures. The 12 and 10 mers had shorten fibril lengths, while the 16 mer contained fibrils and misfolded aggregates, none of the latter were found with the 14 mer.

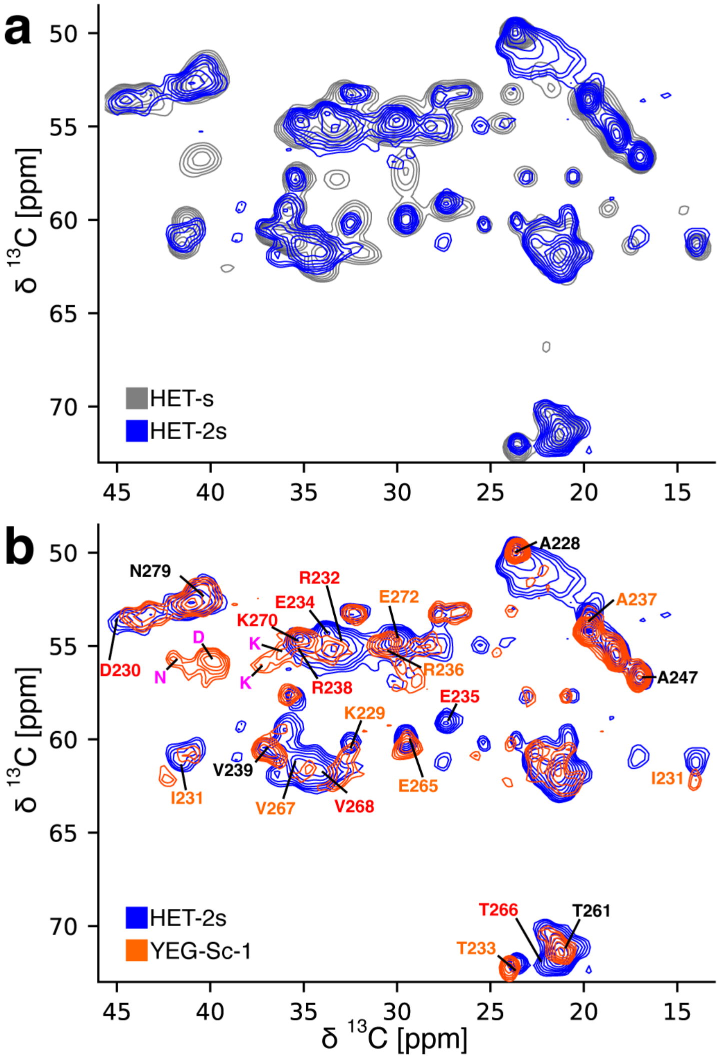

Fig. S2 Separated ssNMR spectra. **a** Overlay of the aliphatic region of 2D ^{13}C-^{13}C DREAM spectra of HET-s (gray) and HET-2s (blue). The almost perfect overlap of these spectra indicates that duplicating the HET-s prion domain sequence does not affect the fibril structure. **b** Overlay of the aliphatic region of 2D ^{13}C-^{13}C DREAM spectra of HET-2s (blue) and YEG-Sc-1 (orange). Residues that were modified (and are now missing) are labeled in red. Residues adjacent to modifications are labeled in orange, these can be detected but with slightly altered peak position. Residues not close in sequence to an insertion site are labeled in black, all of these peaks are unchanged indicating that the overall fold of YEG-Sc-1 is the same as for HET-s and HET-2s.

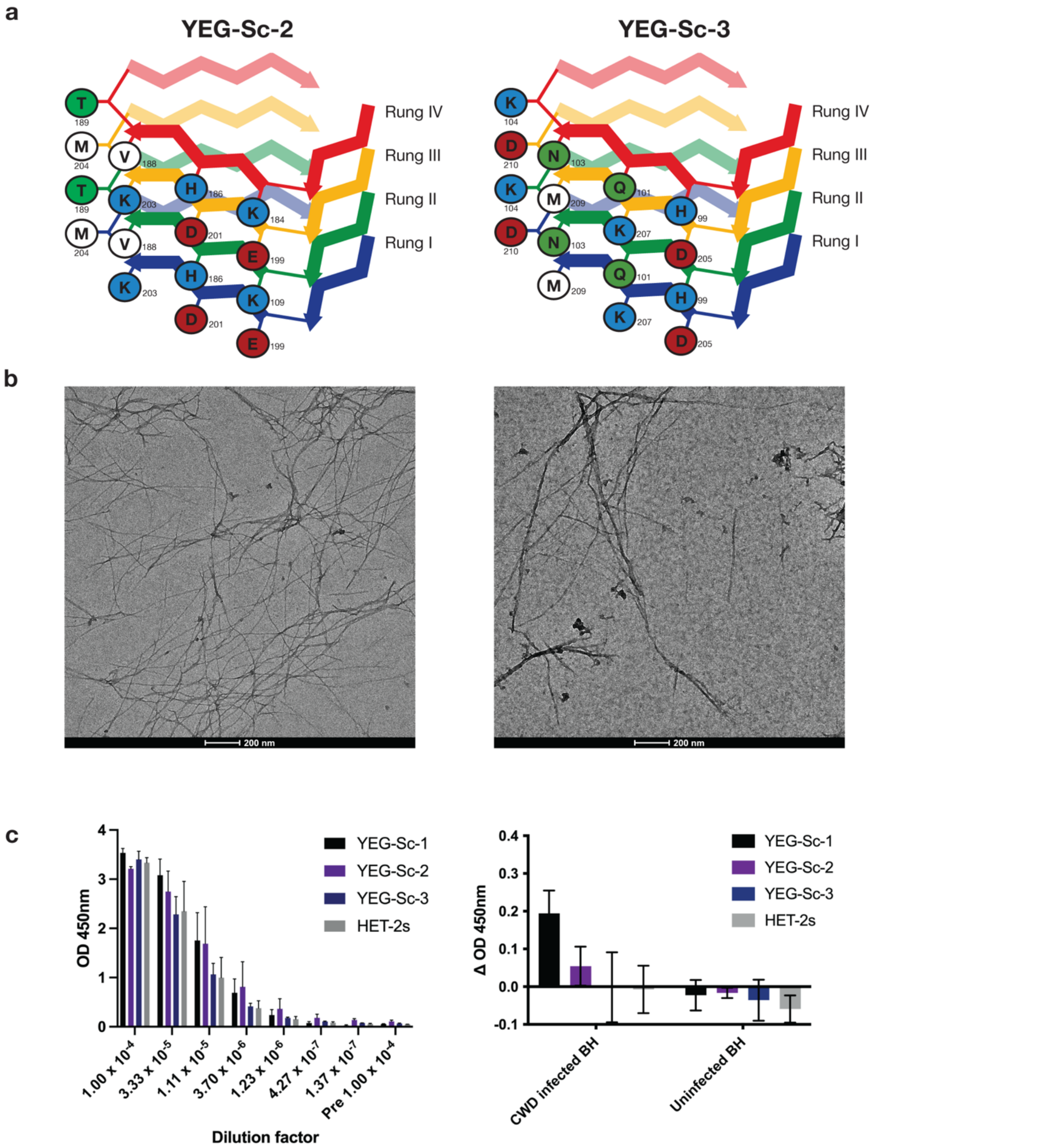

Fig. S3 Characterization of vaccine candidates YEG-Sc-2 and YEG-Sc-3. **a** Cartoons depicting the PrP surface residues inserted to generate YEG-Sc-2 and -3. **b** Electron micrographs (recorded at 19K magnification) of the vaccine candidates positively stained with 1% uranyl acetate. **c** The immune responses of the vaccine candidates in an indirect ELISA (left) and the specificity of the immune responses in a competition ELISA (right). Only YEG-Sc-1 could reliably distinguish between prion infected (CWD) and uninfected brain homogenates.

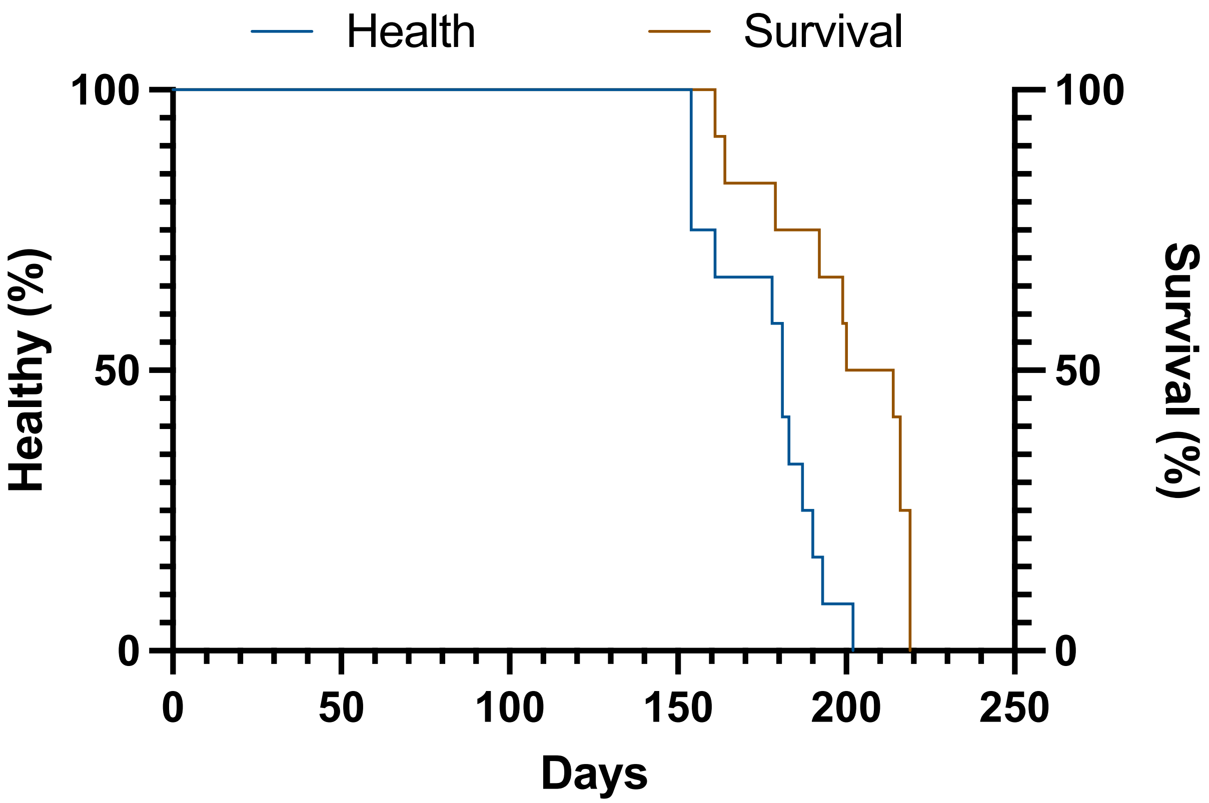

Fig. S4 GSS symptom onset and survival of TgP101L mice. Overlayed Kaplan-Meier curves depicting health (blue, left y-axis) and survival (brown, right y-axis) of mice in days. The onset of symptoms is 177±17 days and survival is 200±22 days (mean ± SD).

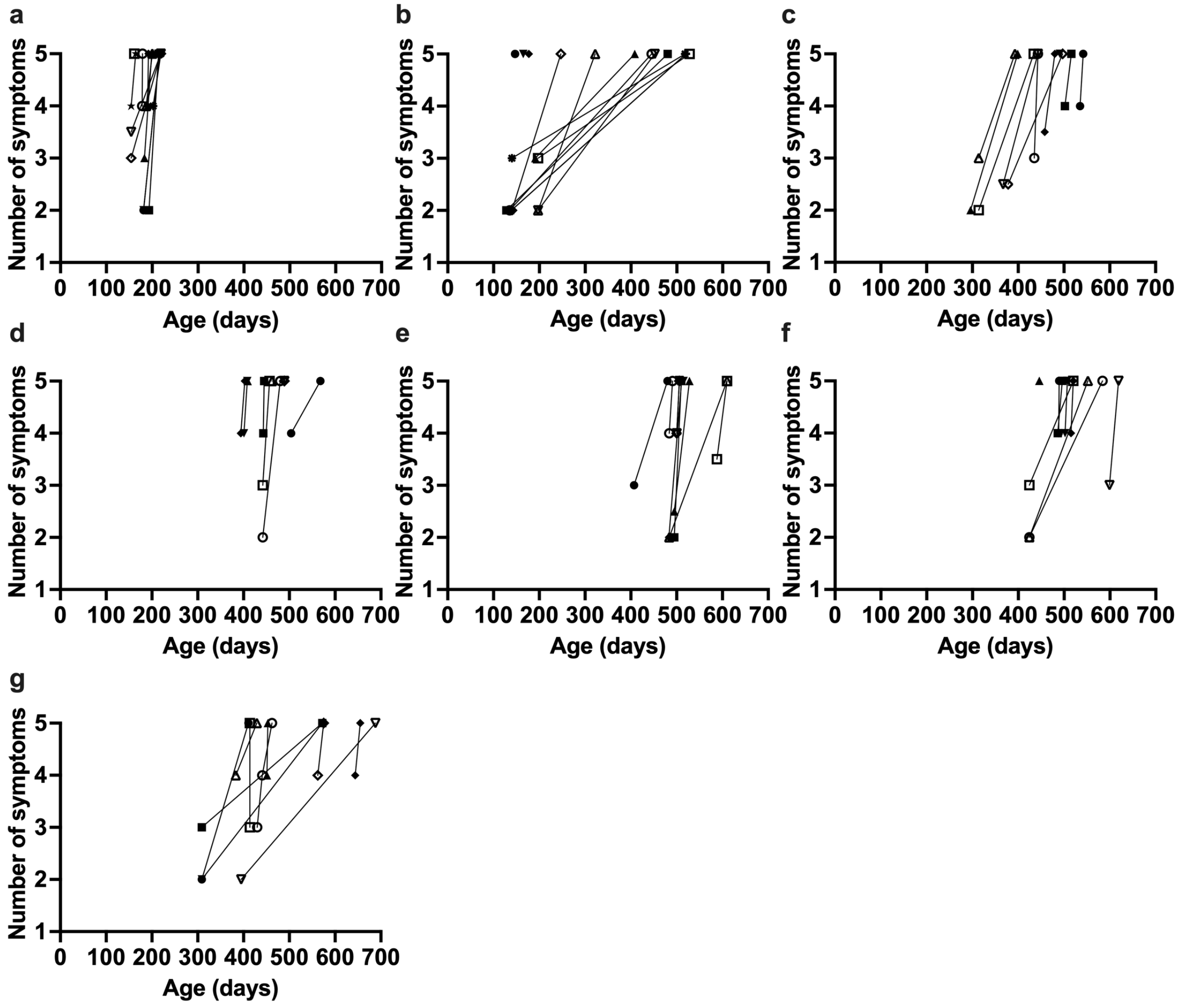

Fig. S5 GSS symptom scoring of TgP101L mice. Progression of disease symptoms from initial onset and terminal disease. Initial onset is defined when 2 or more symptoms were observed and progressing, while terminal disease was defined when 5 or more symptoms were present (see disease evaluation in materials and methods for list of symptoms). A-G is as follows: (A) unimmunized, (B) HET-2s/Freund's, (C) YEG-Sc-1/no adjuvant, (D) YEG-Sc-1/Freund's, (E) YEG-Sc-1/Alum, (F) YEG-Sc-1/QS21, (G) YEG-Sc-1/Alum continuous. Each line and its corresponding symbol represents 1 animal.

```
murine_P101L/1-254     1 --MANLGYWLLALFVTMWTDVGLCKKRPKP-GGWNTGGSRYPGQGSPGGNRYPPQ-GGTWGQ  58
PrPSc106/1-106           --------------------------------------------------------------
murine/1-254           1 --MANLGYWLLALFVTMWTDVGLCKKRPKP-GGWNTGGSRYPGQGSPGGNRYPPQ-GGTWGQ  58
human/1-253            1 --MANLGCWMLVLFVATWSDLGLCKKRPKP-GGWNTGGSRYPGQGSPGGNRYPPQGGGGWGQ  59
cervid/1-256           1 MVKSHIGSWILVLFVAMWSDVGLCKKRPKPGGGWNTGGSRYPGQGSPGGNRYPPQGGGGWGQ  62
bovine/1-264           1 MVKSHIGSWILVLFVAMWSDVGLCKKRPKPGGGWNTGGSRYPGQGSPGGNRYPPQGGGGWGQ  62

murine_P101L/1-254    59 PHGGGWGQPHGGSWGQPHGGSWGQPH-GGGWGQG--------GGTHNQWNKLSKPKTNLKHV 111
PrPSc106/1-106         1 -------------------------------GQG--------GGTHNQWNKPSKPKTNLKHV  23
murine/1-254          59 PHGGGWGQPHGGSWGQPHGGSWGQPH-GGGWGQG--------GGTHNQWNKPSKPKTNLKHV 111
human/1-253           60 PHGGGWGQPHGGGWGQPHGGGWGQPH-GGGWGQG--------GGTHSQWNKPSKPKTNMKHM 112
cervid/1-256          63 PHGGGWGQPHGGGWGQPHGGGWGQPHGGGGWGQ---------GGTHSQWNKPSKPKTNMKHV 115
bovine/1-264          63 PHGGGWGQPHGGGWGQPHGGGWGQPH-GGGWGQPHGGGGWGQGGTHGQWNKPSKPKTNMKHV 123

murine_P101L/1-254   112 AGAAAAGAVVGGLGGYMLGSAMSRPMIHFGNDWEDRYYRENMYRYPNQVYYRPVDQYSNQNN 173
PrPSc106/1-106        24 AGAAAAGAVVGGLGGYMLGSAMSRPMIHF---------------------------------  52
murine/1-254         112 AGAAAAGAVVGGLGGYMLGSAMSRPMIHFGNDWEDRYYRENMYRYPNQVYYRPVDQYSNQNN 173
human/1-253          113 AGAAAAGAVVGGLGGYMLGSAMSRPIIHFGSDYEDRYYRENMHRYPNQVYYRPMDEYSNQNN 174
cervid/1-256         116 AGAAAAGAVVGGLGGYMLGSAMSRPLIHFGNDYEDRYYRENMYRYPNQVYYRPVDQYNNQNT 177
bovine/1-264         124 AGAAAAGAVVGGLGGYMLGSAMSRPLIHFGSDYEDRYYRENMHRYPNQVYYRPVDQYSNQNN 185

murine_P101L/1-254   174 FVHDCVNITIKQHTVTTTTKGENFTETDVKMMERVVEQMCVTQYQKESQAYYDGRRSSSTVL 235
PrPSc106/1-106        53 ---DCVNITIKQHTVTTTTKGENFTETDVKMMERVVEQMCVTQYQKESQAYYDGRRS----- 106
murine/1-254         174 FVHDCVNITIKQHTVTTTTKGENFTETDVKMMERVVEQMCVTQYQKESQAYYDGRRSSSTVL 235
human/1-253          175 FVHDCVNITIKQHTVTTTTKGENFTETDVKMMERVVEQMCITQYERESQAYY--QRGSSMVL 234
cervid/1-256         178 FVHDCVNITVKQHTVTTTTKGENFTETDIKMMERVVEQMCITQYQRESQAYY--QRGASVIL 237
bovine/1-264         186 FVHDCVNITVKEHTVTTTTKGENFTETDIKMMERVVEQMCITQYQRESQAYY--QRGASVIL 245

murine_P101L/1-254   236 FSSPPVILLISFLIFLIVG                                            254
PrPSc106/1-106           -------------------
murine/1-254         236 FSSPPVILLISFLIFLIVG                                            254
human/1-253          235 FSSPPVILLISFLIFLIVG                                            253
cervid/1-256         238 FSSPPVILLISFLIFLIVG                                            256
bovine/1-264         246 FSSPPVILLISFLIFLIVG                                            264
```

Fig. S6 Sequence alignment of prion protein in different species. Sequences of species tested using G1, with histidine and aspartate highlighted in green and teal, respectively, following the same coloring convention as the rest of the figures. Both resides are found in all species except PrP^{Sc}106, which lacks the aspartate. CLUSTAL multiple sequence alignment was performed by MUSCLE 3.8 and visualized with Jalview.

Table S1 List of model-based vaccine candidates and their sequences and properties.

Name	Rung I	Rung II	Rung III	Rung IV	Fibril formation	Folding strategy	Prion specificity after immunization
HET-2s	NSAKDIRTEE	NSVETVVGKG	same as rung I	same as rung II	yes	N/A	no
Non-fibrillar 1	NSATHIQTNK	NNVYEVRGYR	NVAYRIVTQY	NTVTDVKGME	no	none	N/A
Non-fibrillar 2	NSATNIKTVA	NNVYEVRGYR	NVAYRIVTQY	NNVTDVDGKM	no	none	N/A
Non-fibrillar 3	NSATNIKTVA	NDVEDVYGRD	NVAYRIVTQY	NTVIKVMGRV	no	none	N/A
Non-fibrillar 4	NSAKDIRTEE	NSVKTVMGHV	NSADYIDTYY	NSVETVVGKG	no	change middle two rungs	N/A
YEG-Sc-1	NSAKYIDTED	NSVEKVNGKH	same as rung I	same as rung II	yes	repeating rungs I & II	yes
YEG-Sc-2	NSAEDIKTME	NSVKHVQGNK	same as rung I	same as rung II	yes	repeating rungs I & II	no
YEG-Sc-3	NSAEEIDTKM	NSVKKVHGVT	same as rung I	same as rung II	yes	repeating rungs I & II	no

Underlined residues indicate surface exposure

Table S2 Statistics overview of health and survival efficacy data.

	Health			
**Experimental group**	**Group size (n)**	**Disease onset (days)***	**Significance vs. unimmunized mice**	**Significance vs. HET-2s/Freund's mice**
Unimmunized	12	177±17	n/a	$p = 0.5053$
HET-2s/Freund's	13	161±27	$p = 0.5053$	n/a
YEG-Sc-1/No adjuvant	10	412±88	$p < 0.0001$	$p < 0.0001$
YEG-Sc-1/Freund's	10	448±39	$p < 0.0001$	$p < 0.0001$
YEG-Sc-1/QS-21	9	479±58	$p < 0.0001$	$p < 0.0001$
YEG-Sc-1/Alum	10	506±52	$p < 0.0001$	$p < 0.0001$

	Survival				
**Experimental group**	**Group size (n)**	**Total survival (days)*†**	**Disease duration (days)*‡**	**Significance vs. unimmunized mice§**	**Significance vs. HET-2s/Freund's mice§**
Unimmunized	12	200±22	23±23	n/a	$p = 0.0009$
HET-2s/Freund's	13	379±148	218±154	$p = 0.0009$	n/a
YEG-Sc-1/No adjuvant	10	438±100	55±49	$p < 0.0001$	$p = 0.7961$
YEG-Sc-1/Freund's	10	460±50	14±21	$p < 0.0001$	$p = 0.6565$
YEG-Sc-1/QS-21	9	509±73	47±63	$p < 0.0001$	$p = 0.0364$
YEG-Sc-1/Alum	10	527±46	32±39	$p < 0.0001$	$p = 0.0403$

*Average and standard deviation

†Counting from date of birth

‡Disease duration = total duration - disease onset

§Total survival comparison

Table S3 Statistics overview of health and survival efficacy of alum and alum continuous treatment.

	Health		
**Experimental group**	**Group size (n)**	**Disease onset (days)***	**Significance vs. unimmunized mice**
Unimmunized	12	177±17	n/a
YEG-Sc-1/Alum	10	506±52	$p < 0.0001$
YEG-Sc-1/Alum continuous	10	421±111	$p < 0.0001$

	Survival		
**Experimental group**	**Group size (n)**	**Total duration (days)*†**	**Significance vs. unimmunized mice**
Unimmunized	12	200±22	n/a
YEG-Sc-1/Alum	10	527±46	$p < 0.0001$
YEG-Sc-1/Alum continuous	10	523±102	$p < 0.0001$

*Average and standard deviation

†Counting from date of birth
